# Histologic subtyping in pathologic stage I-IIA lung adenocarcinoma provides risk-based stratification for surveillance

**DOI:** 10.18632/oncotarget.26285

**Published:** 2018-11-06

**Authors:** Yusuke Takahashi, Takashi Eguchi, Koji Kameda, Shaohua Lu, Raj G. Vaghjiani, Kay See Tan, William D. Travis, David R. Jones, Prasad S. Adusumilli

**Affiliations:** ^1^ Thoracic Service, Department of Surgery, Memorial Sloan Kettering Cancer Center, New York, NY, USA; ^2^ Department of General Thoracic Surgery, Keio University School of Medicine, Tokyo, Japan; ^3^ Division of Thoracic Surgery, Department of Surgery, Shinshu University, Matsumoto, Japan; ^4^ Department of Thoracic Surgery, National Defense Medical College, Tokorozawa, Japan; ^5^ Department of Pathology, Memorial Sloan Kettering Cancer Center, New York, NY, USA; ^6^ Department of Pathology, Zhongshan Hospital, Fudan University, Shanghai, China; ^7^ Department of Epidemiology and Biostatistics, Memorial Sloan Kettering Cancer Center, New York, NY, USA; ^8^ Center for Cell Engineering, Memorial Sloan Kettering Cancer Center, New York, NY, USA

**Keywords:** recurrence hazard, dynamics, solid, micropapillary, prognosis

## Abstract

**Background:**

We hypothesize that recurrence hazard following resection for stage I-IIA lung adenocarcinoma (ADC) varies according to histologic subtype, which may provide risk stratification for surveillance better than the current uniform follow-up protocol.

**Results:**

Presence (≥5%) of high-grade histologic subtypes (MIP and/or SOL) was associated with a significantly higher recurrence hazard: (1) presence of either MIP or SOL was associated with a significant increase in recurrence hazard during the first two years after surgery; (2) presence of SOL was associated with an increase in recurrence hazard—in particular, distant recurrence hazard—during the first year after surgery; (3) absence of high-grade subtypes (515/1,572 patients) was associated with a very low recurrence hazard (<2% risk/year) during the first ten years after surgery.

**Methods:**

All hematoxylin and eosin–stained tumor slides from pathologic stage I-IIA lung ADC (*n* = 1572) were reviewed for quantification of the percentage of each histological subtype. Recurrence hazard was estimated using the Kernel-Epanechnikov smoothing procedure. The association between recurrence hazard and high-grade histologic subtypes (micropapillary [MIP] and solid [SOL]) was assessed.

**Conclusions:**

Our findings suggest that histologic subtyping has utility for identifying recurrence hazard for surgically resected stage I-IIA lung ADC patients and provide rationale for establishing risk-based surveillance.

## INTRODUCTION

Complete resection offers the best possibility of cure for patients with early-stage non-small cell lung cancer (NSCLC) and appropriately selected patients with locally advanced NSCLC [1]. However, the incidence of recurrence for resected NSCLC ranges from 15% to 25%, even with curative-intent resection in patients with stage I NSCLC [1–3]. Whereas detecting recurrence early may allow for potential treatment [4, 5] and avoid complications that compromise quality of life, detection involves significant effort and resources for both the patient and the medical establishment. National practice guidelines for stage I NSCLC recommend intensive surveillance during the first two years after surgery [4–7], as the recurrence hazard during this period has been reported to be much higher than in later years [8, 9]. Although outcomes following surgery may vary between cure or early or late recurrence, individualized risk stratification for rational surveillance is not currently available [10–13].

Lung adenocarcinoma (ADC) is the most common histologic type of NSCLC. In the 2015 World Health Organization (WHO) Classification of Tumors of the Lung, Pleura, Thymus and Heart [14], lung ADC was characterized as a heterogeneous mixture of histologic subtypes. Investigators, including from our group, have documented the prognostic and therapeutic significance of histologic subtyping—in particular, the poor prognostic correlation with micropapillary (MIP) and solid (SOL) patterns, especially in early-stage lung ADC [15–17, 12, 18, 19].

We hypothesize that recurrence hazard following resection for stage I-IIA lung ADC varies according to histologic subtype and that this variation in hazard may provide risk stratification for surveillance of these patients.

## RESULTS

### Clinicopathologic characteristics and recurrent disease

**Figure 1:**
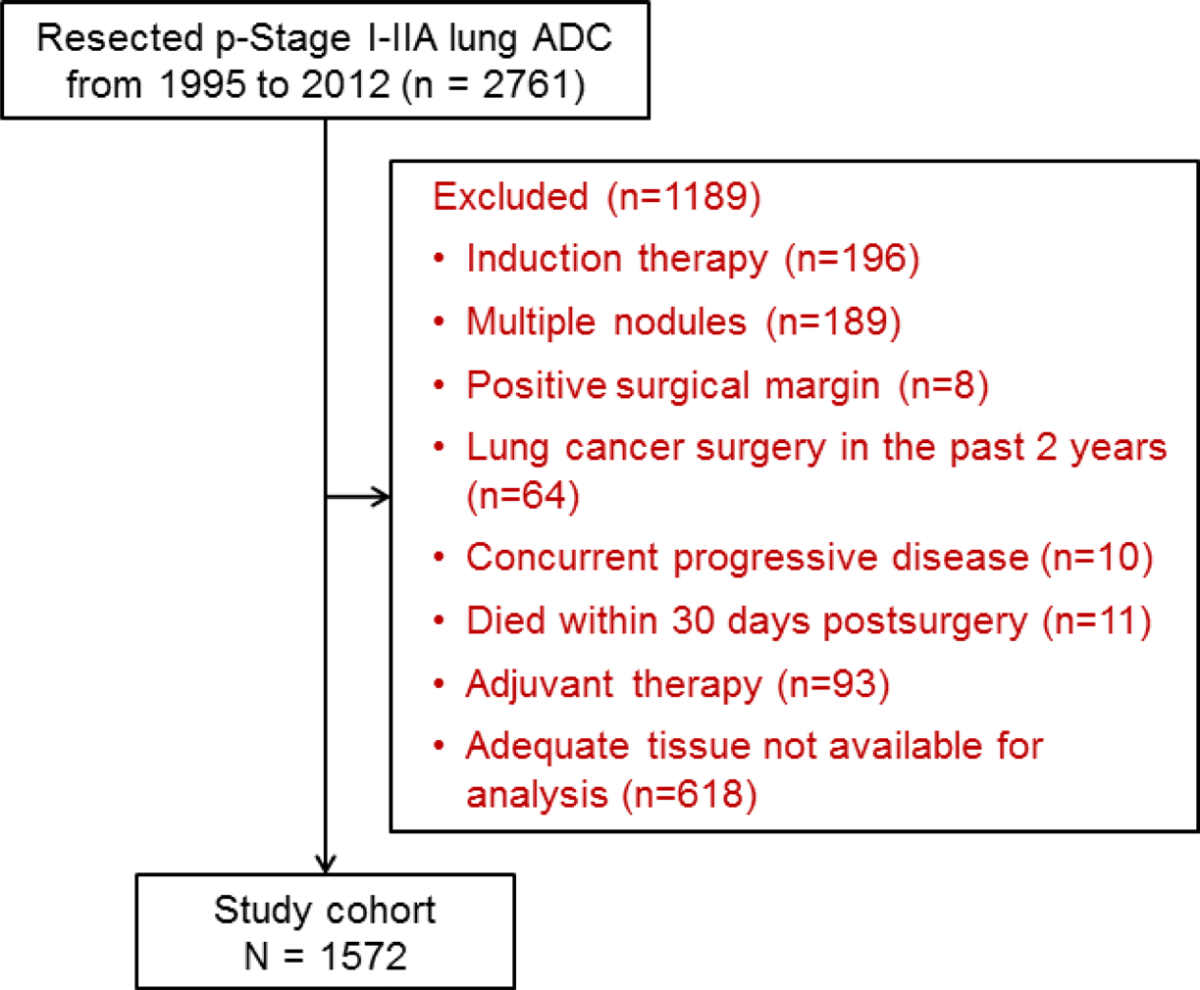
CONSORT diagram ADC = adenocarcinoma; p-Stage = pathologic stage.

The study cohort included 1,572 patients (Figure 1). Demographic and clinicopathological information is listed in Table 1. Presence (≥5%) of MIP subtype was identified in 737 tumors (47%), presence of SOL subtype was identified in 640 tumors (41%), and presence of both MIP and SOL subtypes was identified in 320 tumors (20%). LEP was the predominant subtype in 228 tumors (15%), ACI in 642 (41%), PAP in 313 (20%), MIP in 86 (5%), SOL in 235 (15%), MUC in 59 (4%), and COL in 9 (1%). Of the 1,572 cases, 1,159 (74%) were pathologic stage IA, 341 (22%) were IB, and 72 (5%) were IIA. The 5-year cumulative incidence of any recurrence was 20% (95% confidence interval 18–22%); specifically, 5-year cumulative incidence was 10% (8–11%) for locoregional recurrence and 13% (11–15%) for distant recurrence.

**Table 1:** Patient demographics characteristics (*n* = 1,572)

Characteristic	No. (%)
Age at surgery, years, median (IQR)	69.0 (62.0–76.1)
Sex	
Female	979 (62)
Male	593 (38)
Smoking history	
Never	271 (17)
Former	1,103 (70)
Current	198 (13)
Surgical procedure	
Pneumonectomy (including completion)	2 (<1)
Bilobectomy	8 (1)
Lobectomy	1,076 (68)
Segmentectomy	157 (10)
Wedge resection	329 (21)
Combination of high-grade subtypes^a^	
MIP–/SOL–	515 (33)
MIP+/SOL–	417 (27)
MIP–/SOL+	320 (20)
MIP+/SOL+	320 (20)
Predominant histologic subtype	
Lepidic	228 (15)
Acinar	642 (41)
Papillary	313 (20)
Micropapillary	86 (5)
Solid	235 (15)
Invasive mucinous	59 (4)
Colloid	9 (1)
Lymphatic invasion	
Negative	1,056 (67)
Positive	516 (33)
Vascular invasion	
Negative	1,162 (74)
Positive	410 (26)
Pleural invasion	
Negative	1,334 (85)
Positive	238 (15)
STAS (*n* = 1370)	
Negative	871 (64)
Positive	499 (36)
Pathologic stage	
IA	1,159 (74)
IB	341 (22)
IIA	72 (5)

Abbreviations: IQR = interquartile range; MIP = micropapillary; SOL = solid; STAS = spread through air space.

^a^ Absent (–) = <5%; present (+) = ≥5%.

### Analysis of recurrence hazard

**Figure 2:**
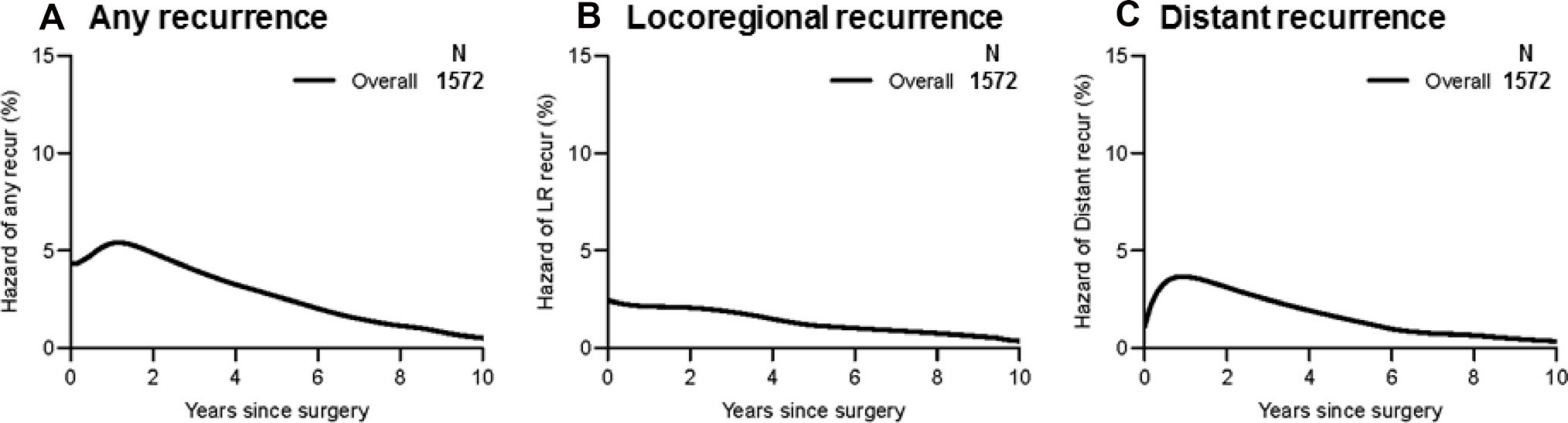
Recurrence hazard curves for overall patients (**A**) The hazard of any recurrence demonstrates a peak at the second year after surgery. It declines thereafter but maintains a certain level. (**B**) The hazard of locoregional recurrence has no evident peak and exhibits a gradual decline. (**C**) The hazard of distant recurrence shows a significant peak at the second year after surgery and a gradual decrease thereafter, which is similar to the hazard of any recurrence.

For all cases, the hazard curve for any recurrence clearly increased up to the second year after surgery, followed by a decrease (Figure 2A); the hazard curve for locoregional recurrence, on the other hand, gradually decreased (Figure 2B). Similar to the curve for any recurrence, the hazard curve for distant recurrence clearly increased up to approximately the second year after surgery then gradually decreased thereafter (Figure 2C).

**Figure 3:**
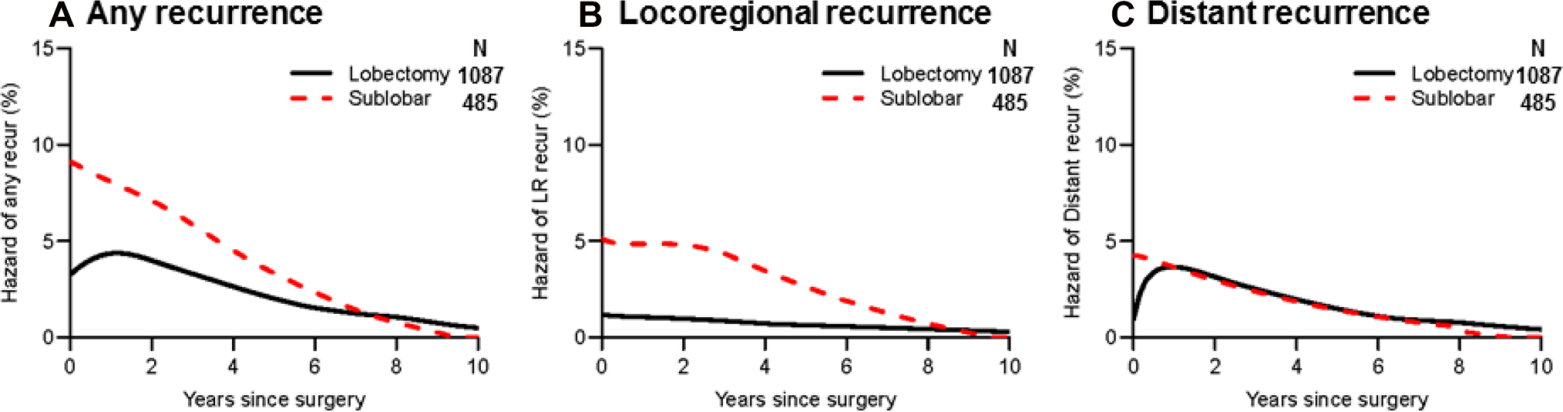
Recurrence hazard curves by surgical procedure (**A**) Hazard curves for any recurrence by surgical procedure are shown. Recurrence hazard was approximately 2-fold higher in the sublobar resection group (black line, *n* = 485) than in the lobar resection group (red broken line, *n* = 1,087) during the first two years after surgery. (**B**) Hazard curves for locoregional recurrence by surgical procedure are shown. Locoregional recurrence hazard was higher in the sublobar resection group than in the lobar resection group; sublobar resection had a constant high locoregional recurrence hazard up to three years after surgery. (**C**) Hazard curves for distant recurrence by surgical procedure are shown. Distant recurrence hazard reached a peak during the first year after surgery in the sublobar resection group; it peaked later in the lobectomy group.

To investigate the influence of surgical procedure on recurrence hazard, we compared recurrence hazard among patients who had undergone lobectomy with that among patients who had undergone sublobar resection (segmentectomy or wedge resection). As shown in Figure 3A, recurrence hazard for sublobar resection was approximately 2-fold higher than that for lobar resection up to four years after surgery. In particular, locoregional recurrence hazard was much higher in the sublobar resection group than in the lobar resection group, which had a grossly constant recurrence hazard (Figure 3B). Distant recurrence hazard peaked early during the first year in the sublobar resection group and later during the first year in the lobectomy group. Thereafter, the curves for both groups declined in a similar fashion (Figure 3C).

### Impact of high-grade histologic subtypes on recurrence hazard

**Figure 4:**
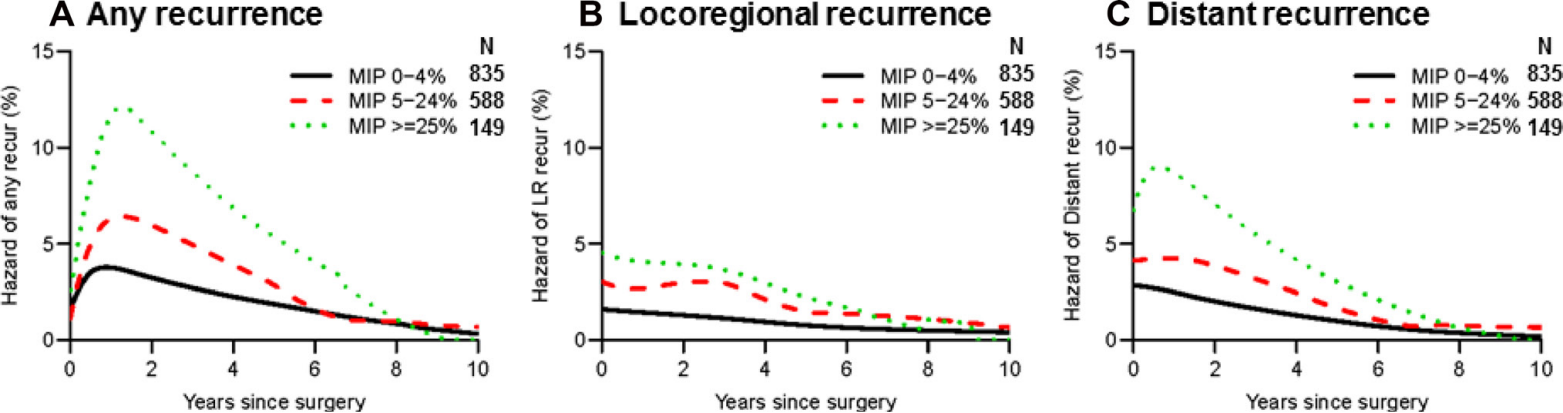
Recurrence hazard curves by percentage of micropapillary (MIP) subtype (**A**) Hazard curves for any recurrence by MIP percentage are shown. The MIP ≥25% group (green dotted line, *n* = 149) had the highest hazard of any recurrence within the 5-year period after surgery, followed by the 5%–24% group (red broken line, *n* = 588) and the 0%–4% group (black line, *n* = 835). (**B**) Hazard curves for locoregional recurrence by MIP percentage are shown. The differences between the groups were smaller than those for any or distant recurrence. (**C**) Hazard curves for distant recurrence by MIP percentage are shown. The differences between groups were greater than those for locoregional recurrence.

We investigated the impact of histologic subtype on recurrence hazard after complete resection. We first focused on the association between percentage of MIP subtype and recurrence hazard. As shown in Figure 4A, hazard of any recurrence was well-stratified by percentage of MIP subtype (MIP 0%-4% vs. 5%-24% vs. ≥25%). The figure clearly shows that recurrence hazard increased and the peak became higher as the percentage of MIP subtype increased. Although patients without presence of MIP subtype (0%–4%) had a lower recurrence hazard than patients with presence of MIP subtype (5%–24% and ≥25%), recurrence hazard for all groups was highest during the first two years after surgery, before gradually decreasing thereafter. Although locoregional recurrence hazard differed according to the percentage of MIP subtype, the difference between groups was much smaller than for distant recurrence (Figure 4B and 4C).

**Figure 5:**
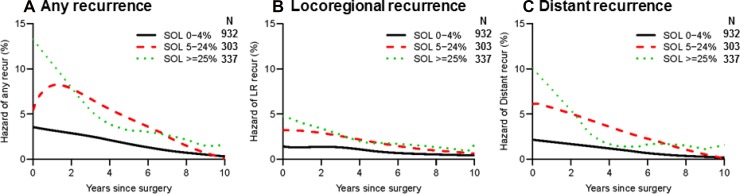
Recurrence hazard curves by percentage of solid (SOL) subtype (**A**) Hazard curves for any recurrence by SOL percentage are shown. Recurrence hazard was lowest for the 0%-4% group (black line, *n* = 932). Although the ≥25% group (green dotted line, *n* = 337) had the highest peak (>10%) during the first year after surgery, the hazard for this group declined sharply thereafter. The 5%–24% group (red broken line, *n* = 303) had a significant peak at the second year after surgery. (**B**) Hazard curves for locoregional recurrence by SOL percentage are shown. Locoregional recurrence hazard was lowest in the 0%–4% group. (**C**) Hazard curves for distant recurrence by SOL percentage are shown. Distant recurrence hazard peaked during the first year after surgery in the ≥25% and 5%–24% groups; it was lowest in the 0%–4% group.

We next looked at the association between percentage of SOL subtype (0%–4% vs. 5%–24% vs. ≥25%) and recurrence hazard. Hazard of any recurrence was higher in the ≥25% group than in the 5%-24% group during the first year after surgery (Figure 5A). The 0%-4% group had the lowest hazard of any recurrence, which gradually declined from baseline. Locoregional recurrence hazard was higher in the ≥25% group than in the 5%–24% group during the first two years after surgery; after this point, the curves for both groups declined in a similar fashion (Figure 5B). Distant recurrence hazard was higher in the ≥25% group than in the 5%–24% group during the first two years; after this point, the hazard for the ≥25% group fell below that for the 5%–24% group, up until approximately year 7 (Figure 5C).

**Figure 6:**
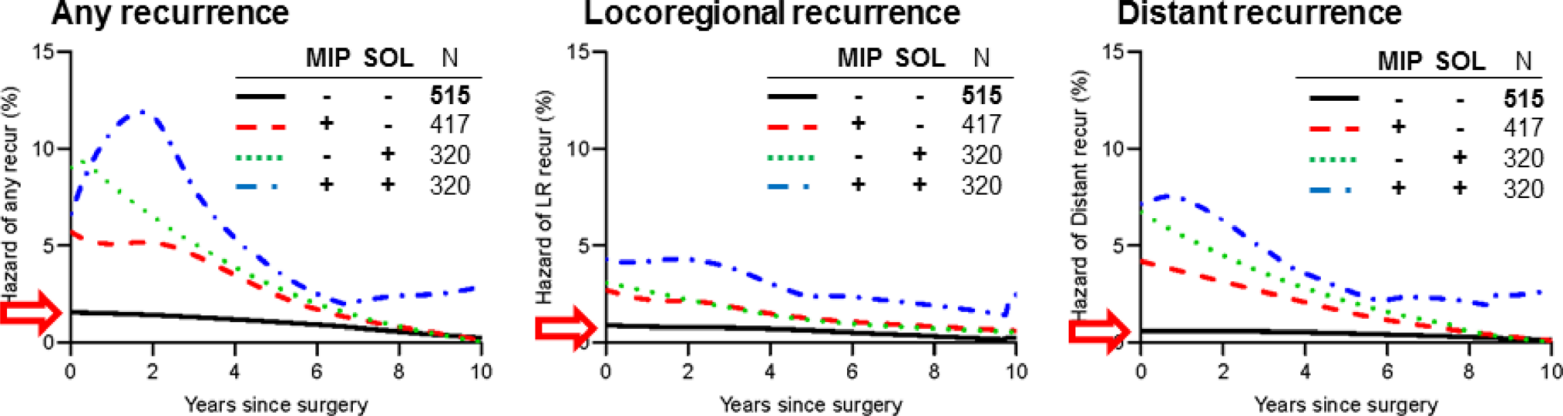
Recurrence hazard curves by presence (≥5%) or absence (<5%) of both micropapillary (MIP) and solid (SOL) subtypes Patients were stratified into four groups: both MIP and SOL absent (MIP–/SOL–), only MIP present (MIP+/SOL–), only SOL present (MIP–/SOL+), and both MIP and SOL present (MIP+/SOL+). (**A**) Hazard curves for any recurrence by MIP-SOL combination are shown. Hazard of any recurrence was highest in the MIP+/SOL+ group (blue broken line, *n* = 320)—with a significant increase (>10%) in recurrence hazard during the first two years—except for during the first year after surgery, when hazard of any recurrence was higher in the MIP–/SOL+ group (red broken line, *n* = 320). Hazard of any recurrence was lowest in the MIP–/SOL– group (black line, *n* = 515) and consistently <2% during the ten years after surgery, without any significant increase in hazard (red arrow). (**B**) Hazard curves for locoregional recurrence by MIP-SOL combination are shown. Locoregional recurrence hazard was highest in the MIP+/SOL+ group, followed by MIP+/SOL–, MIP–/SOL+, and MIP–/SOL–. The SOL 5%–24% and SOL ≥25% groups had similar dynamics. The MIP–/SOL– group consistently had the lowest locoregional recurrence hazard, without any increases (red arrow). (**C**) Hazard curves for distant recurrence by MIP-SOL combination are shown. The MIP–/SOL– group consistently had the lowest distant recurrence hazard, without any increases (red arrow); the other groups peaked during the first year.

Because the presence of even a small amount (5%–24%) of MIP and SOL subtypes increases hazard of recurrence [12, 20], we stratified patients into four groups on the basis of the presence (≥5%) or absence (<5%) of both subtypes: both MIP and SOL absent (MIP–/SOL–), only MIP present (MIP+/SOL–), only SOL present (MIP–/SOL+), and both MIP and SOL present (MIP+/SOL+). Hazard of any recurrence was highest in the MIP+/SOL+ group—with a significant increase (>10%) in recurrence hazard during the first two years—except for during the first year after surgery, when hazard of any recurrence was higher in the MIP-/SOL+ group (Figure 6A). Hazard of any recurrence increased early during the first year in the MIP–/SOL+ group, before declining thereafter. In the MIP+/SOL– group, hazard of any recurrence increased between year 1 and 3, before declining thereafter. Of note, in the MIP–/SOL– group, hazard of any recurrence was consistently <2% during the ten years after surgery, without any evident increases. Similarly, hazard of locoregional and distant recurrence were lowest in the MIP–/SOL– group, without any increases (Figure 6B and 6C).

## DISCUSSION

Our study demonstrates that, for resected pathologic stage I-IIA lung ADC, (1) recurrence hazard was clearly stratified by presence of high-grade histologic subtypes (MIP, SOL); (2) presence of high-grade histologic subtypes was associated with a significant increase in recurrence hazard during the first two years after surgery; (3) presence of SOL subtype was associated with a significant increase in hazard of recurrence—in particular, distant recurrence—during the first year after surgery; and (4) recurrence hazard in patients with no high-grade subtypes (MIP–/SOL–; one-third of patients) was consistently very low (<2% risk/year) during the ten years after surgery, without any evident increases. These findings may provide a rationale for prospectively investigating the utility of modifying existing risk-based surveillance protocols. To the best of our knowledge, this is the first report to demonstrate the influence of histologic subtype on recurrence hazard using a large series of patients with pathologic stage I-IIA lung ADC.

Current surveillance protocols include uniformly intensive surveillance during the first two years after surgery [4–7]. However, there is a paucity of evidence to support these recommendations. In particular, there have been no studies to date that have directly demonstrated a survival benefit associated with follow-up surveillance. Only one meta-analysis demonstrated that detection of asymptomatic recurrence was associated with better survival, whereas intensive follow-up was not [21]. The authors noted that the small studies they included were likely underpowered to show survival differences by intensive follow-up, while better survival with asymptomatic recurrence was affected by lead-time bias [21].

Yamauchi *et al.* reported that, among patients with resected NSCLC, the risk of recurrence was highest during the first two years after surgery and that recurrence hazard for patients with ADC was higher than for patients with squamous cell carcinoma [22]. Postresection recurrence is affected by many factors, including tumor aggressiveness [23], burden of remaining microscopic cancer cells [24], and the host-tumor immune microenvironment [13]. Our previous studies have shown that lung ADC histologic subtype is not only a significant risk factor for recurrence in patients with resected early-stage lung ADC [25, 26] but is also associated with molecular features [27], gene mutation status [28], and the immune microenvironment [13, 29]. Therefore, it is not surprising that our findings demonstrate well-stratified recurrence dynamics by histologic subtype.

Our findings further confirm the validity of stratification using histologic subtype, even among patients with pathologic stage I-IIA lung ADC. We observed a strong association between presence of high-grade subtypes and high hazard of recurrence (>10% each year during the first two years after surgery)—this supports the recommendation for intensive follow-up surveillance during the first two years after surgery, particularly for patients with high-grade tumors. Patients with high-grade tumors have much higher recurrence hazards during the first two years after surgery, with more-frequent distant recurrences than in later years. Therefore, surveillance examinations that cover distant sites can be prioritized for these high-risk patients during intensive follow-up in the first two years after surgery.

Our findings—based on a retrospective analysis of a large cohort of patients—provide rationale for future prospective investigation. Patients without high-grade subtypes had a consistently low recurrence hazard (<2%/year) during the ten years after surgery, without any significant increases in recurrence hazard. Forgoing intensive follow-up for patients without high-grade subtypes may be an acceptable option. Such a strategy, however, necessitates thorough histologic sampling and evaluation of resected tumors, as recommended in the 2015 WHO classification, to ensure that high-grade histologic subtypes are not present.

## MATERIALS AND METHODS

### Patients

This retrospective study was approved by the Institutional Review Board (#WA269-08) at Memorial Sloan Kettering Cancer Center (MSK). We performed a retrospective review of the prospectively maintained MSK Thoracic Surgery Service Lung Cancer Database for patients treated between January 1995 and December 2012. Inclusion criteria were diagnosis of pathologic stage I-IIA lung ADC and hematoxylin and eosin (H&E)–stained slides available for pathologic review. Staging was determined in accordance with the eighth edition of the American Joint Committee on Cancer staging manual [30]. Exclusion criteria were previous induction therapy or adjuvant therapy, presence of multiple nodules, presence of tumors with positive margins, lung cancer surgery within the past two years, and concurrent progressive diseases other than lung cancer. We also excluded 11 patients who died within 30 days after surgery (7 patients who underwent lobectomy and 4 patients who underwent sublobar resection) (Figure 1).

Postoperative surveillance was performed in accordance with the National Comprehensive Cancer Network guidelines [6]. Each patient received a physical examination, interval history, and chest/upper abdominal computed tomography (CT) scan every six to twelve months during the first two years after resection and yearly thereafter. Patients were monitored by either the thoracic surgeon or a nurse practitioner trained in thoracic survivorship care at MSK. Recurrences were confirmed by cytologic or histologic evaluation and were classified in accordance with the Society of Thoracic Surgeons Workforce recommendations [31]. Loco-regional recurrence included either local or regional recurrence or both. Local recurrence was defined by evidence of a tumor in the same lobe or at the surgical margin of the original tumor. Regional recurrence was defined by evidence of a tumor in the ipsilateral lobe, in the ipsilateral hilar lymph nodes (N1), or in the ipsilateral mediastinal lymph nodes (N2). Distant recurrence was defined by evidence of a tumor in the contralateral lung, in the contralateral mediastinal or ipsilateral supraclavicular lymph nodes (N3), or outside the hemithorax. In cases where a new tumor developed in the lung, the histologic profile was reviewed to determine whether the new tumor was a metachronous primary tumor or a recurrence, as previously described [32].

### Histopathologic evaluation

All available H&E-stained tumor slides were reviewed by two pathologists who were blinded to patient demographic and clinical information. Any discrepancies between the pathologists were resolved by consensus using a multiheaded microscope. Tumors were classified, in accordance with the WHO classification [14], as adenocarcinoma *in situ* (AIS), minimally invasive adenocarcinoma (MIA), or invasive ADC, which, in turn, was subdivided into lepidic (LEP)–predominant, acinar (ACI)–predominant, papillary (PAP)–predominant, MIP-predominant, SOL-predominant, colloid (COL)–predominant, and invasive mucinous (MUC) adenocarcinoma. The percentage of each histologic subtype was recorded in 5% increments, and a subtype was considered present if it made up ≥5% of the tumor. In 1370 patients, spread through air spaces was evaluated [33].

### Statistical analysis

Continuous variables are presented as medians with interquartile ranges (IQRs). Time to recurrence was measured from the date of surgery until the first diagnosis of any recurrence (either locoregional or distant). Hazard function estimates were calculated using the Epanechnikov boundary kernel function with both left and right boundary corrections. The time domain minimum was 0; the maximum time required at least 10 patients to remain at risk. The pilot bandwidth used in the mean-square error minimization was the one recommended by Müller and Wang [34]. Cumulative incidence was estimated using the competing risk approach: for any recurrence, death without recurrence is considered a competing risk; for locoregional recurrence, distant recurrence and death without recurrence are considered competing risks; while for distant recurrence, locoregional recurrence and death without recurrence are considered competing risks. All statistical analyses were performed using *R* 3.3.1 (*R* Core Team, Vienna, Austria) with the *muhaz* package. Comparisons between surgical groups and histologic subtypes are descriptive; no formal statistical testing was conducted.

## CONCLUSIONS

This study demonstrates that, for pathologic stage I-IIA lung ADC, recurrence hazard is clearly stratified by histologic subtype, especially by the presence of high-grade subtypes (MIP, SOL). One-third of patients with stage I-IIA lung ADC had no high-grade subtypes; in these patients, recurrence hazard was consistently very low (<2% risk/year) during the ten years after surgery, without any evident increases in hazard. Our findings suggest that histologic subtyping has utility for determining surveillance protocols. For low-risk patients, a reduced frequency of follow-up during the first two years after surgery may be a safe and acceptable option.

## Abbreviations

ACI: acinar
ADC: adenocarcinoma
AIS: adenocarcinoma *in situ*
COL: colloid
CT: computed tomography
H&E: hematoxylin and eosin
IQR: interquartile range
LEP: lepidic
MIA: minimally invasive adenocarcinoma
MIP: micropapillary
MSK: Memorial Sloan Kettering Cancer Center
MUC: invasive mucinous
NSCLC: non-small cell lung cancer
PAP: papillary
PET/CT: positron emission tomography/computed tomography
SOL: solid
WHO: World Health Organization
